# Microstomia Correction in an 11‐Year‐Old Girl With Freeman–Burian Syndrome

**DOI:** 10.1155/crid/2707697

**Published:** 2026-09-15

**Authors:** Leulseged Tilahun, Tewodros Tefera, Anteneh Yifru

**Affiliations:** ^1^ Department of Oral and Maxillofacial Surgery, Yekatit 12 Hospital Medical College, Yekatit 12 Hospital Medical College, Addis Ababa, Ethiopia; ^2^ Department of Anesthesia, Critical Care and Pain Medicine, Yekatit 12 Hospital Medical College, Addis Ababa, Ethiopia

**Keywords:** commissuroplasty, Converse V-Y mucosal advancement, fiber-optic intubation, Freeman–Burian syndrome, microstomia

## Abstract

**Introduction:**

Freeman–Burian syndrome (FBS) is a congenital myopathic craniofacial condition that often includes features outside the craniofacial region. Among the craniofacial manifestations of FBS, the severely reduced oral aperture stands out as one of the most functionally limiting features of the condition. This marked constriction compromises nutritional intake, speech clarity, oral health maintenance, and airway access. Additionally, the distinctive “pursed‐lip/whistling” facial appearance imposes considerable psychological and social challenges.

**Case Presentation:**

An 11‐year‐old girl was evaluated for challenges consuming solid food and prolonged meal duration, issues that had persisted since early childhood. Physical assessment demonstrated characteristic FBS findings meeting the diagnostic criteria, including microstomia (interlabial distance of 24 mm at maximum opening), an H‐shaped chin defect, hypoplastic nasal alae, and prominent nasolabial creases. Additional findings included mild thoracic scoliosis and reduced stature.

**Management:**

The patient underwent bilateral surgical widening of the oral commissures employing Converse′s V‐Y mucosal advancement flap technique to enlarge the restricted mouth opening. Fiber‐optic assisted nasal intubation was utilized for airway management.

**Conclusion:**

Optimal outcomes depend on an accurate and early diagnosis and understanding of the myopathic craniofacial basis and origin of the syndrome, together with an anticipatory and coordinated team‐based strategy. Management must specifically target the fibrous constricting bands, ensure an adequate oral aperture for nutrition and dental care, support long‐term needs of the patient, and incorporate careful preparation for anesthesia‐associated complications.

## 1. Introduction

Microstomia is an abnormally small oral aperture that may result from various etiologies, including congenital defects, trauma, or surgical resections [1, 2]. Congenital microstomia is rare, with the exception of conditions such as epidermolysis bullosa, Freeman–Burian syndrome (FBS), and Kawasaki disease. The major causes of microstomia overall are trauma (mechanical, thermal, or chemical, such as caustic soda burns) and skin cancer excision. Regardless of the etiology, alteration of the circumoral anatomy results in decreased vertical and horizontal dimensions of the mouth opening [2, 3]. These changes can impair pronunciation, speech, swallowing, and food intake; may prevent optimal dental and oral hygiene; and can affect mandibular motion and aesthetic appearance [4, 5].

FBS is a rare congenital autosomal dominant syndrome. Despite its rarity, it is clinically remarkable because of visible deformities of the face and extremities [6, 7]. Although hand and foot deformities are common, they are not required for diagnosis. Microstomia in FBS represents one of functionally debilitating components of the phenotype. It extends beyond simple skin tightness, representing a complex three‐dimensional tissue deficit. This deficit includes rigid, inelastic skin and oral lining; an underdeveloped and fibrous orbicularis oris muscle that creates a sphincter‐like constriction (in some cases, the orbicularis oris may be essentially absent, replaced by cicatricial tissue that continues to contract); and insufficient volume in the subcutaneous and submucosal layers [8, 9].

Treatment of microstomia in FBS is challenging because of both surgical and anesthesia‐related difficulties. The surgical challenge stems from the complex nature of the lip and oral commissure, compounded by tissue contracture and fibrosis that is difficult to manipulate. Anesthesia‐related challenges include a difficult airway. Patients may experience hypermetabolic states resembling malignant hyperthermia (MH) that are stress‐induced and ibuprofen‐ and paracetamol‐susceptible, and an increased risk for postoperative pulmonary complications, such as pneumonia, empyema, respiratory insufficiency, and recurrent respiratory tract infections [6, 10, 11].

This report describes the case of an 11‐year‐old girl with FBS who presented with feeding dysfunction and was treated for microstomia with commissuroplasty.

## 2. Case Report

### 2.1. Case History and Physical Examination

An 11‐year‐old female presented with significant challenges in consuming solid foods and prolonged meal durations since early childhood, as reported by both the patient and her father. Her feeding difficulties have markedly exacerbated in recent years, resulting in jaw fatigue and facial discomfort. She has developed an active aversion to meat products and raw vegetables. There is no history of prior surgical interventions or speech therapy for dysarthria. Additionally, there is no documented family history of analogous issues.

**Figure 1:**
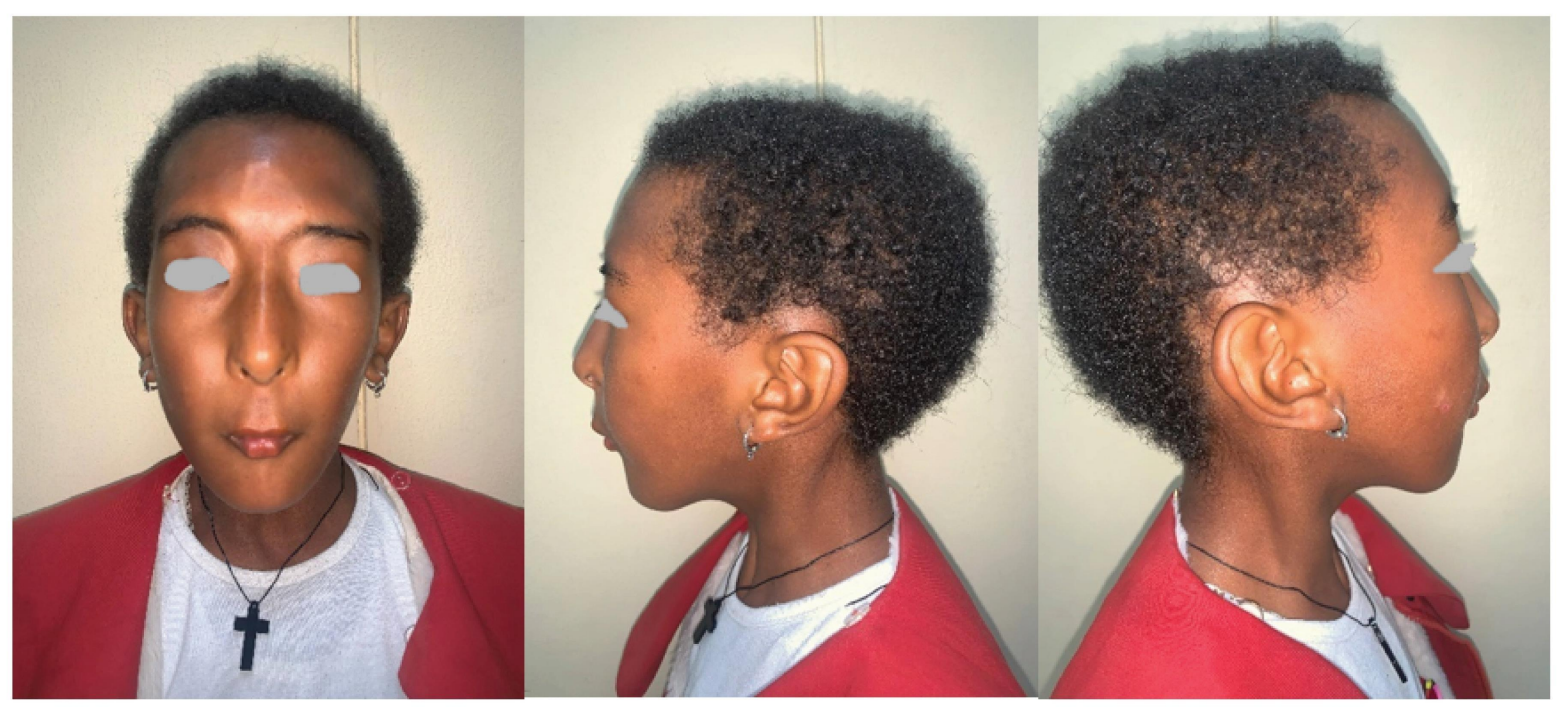
A characteristic whistling facial appearance. A mask‐like facial appearance with tightly pursed lips, microstomia, long philtrum, facial bone flattening, notable epicanthal fold, moderate ptosis, prominent cheeks, and H‐shaped chin dimples.

Physical examination revealed a weight of 20 kg, height of 128 cm, and body mass index of 12.2, indicating underweight and short stature; however, her cognitive development corresponds appropriately to her chronological age. The patient met the craniofacial criteria required for FBS diagnosis, namely a whistling‐face phenotype (pursed lips), microstomia (interlabial distance approximately 24 mm at maximum opening and intercommissural distance approximately 20 mm), hypoplastic nasal alae, prominent nasolabial folds (even in a relaxed state), and an H‐shaped chin dimple. She exhibited additional classic features associated with FBS, including a mask‐like facial appearance, long philtrum, notable epicanthal folds, hypertelorism, moderate ptosis, and low‐set, posteriorly canted ears. Her lengthened face suggests mild craniosynostosis as shown in Figure 1. There is a limited neck range of motion.

Intraoral examination revealed suboptimal oral hygiene, numerous carious lesions, severe dental crowding, a high‐arched palate, significant micrognathia with a V‐shaped mandible, and Class II malocclusion.

**Figure 2:**
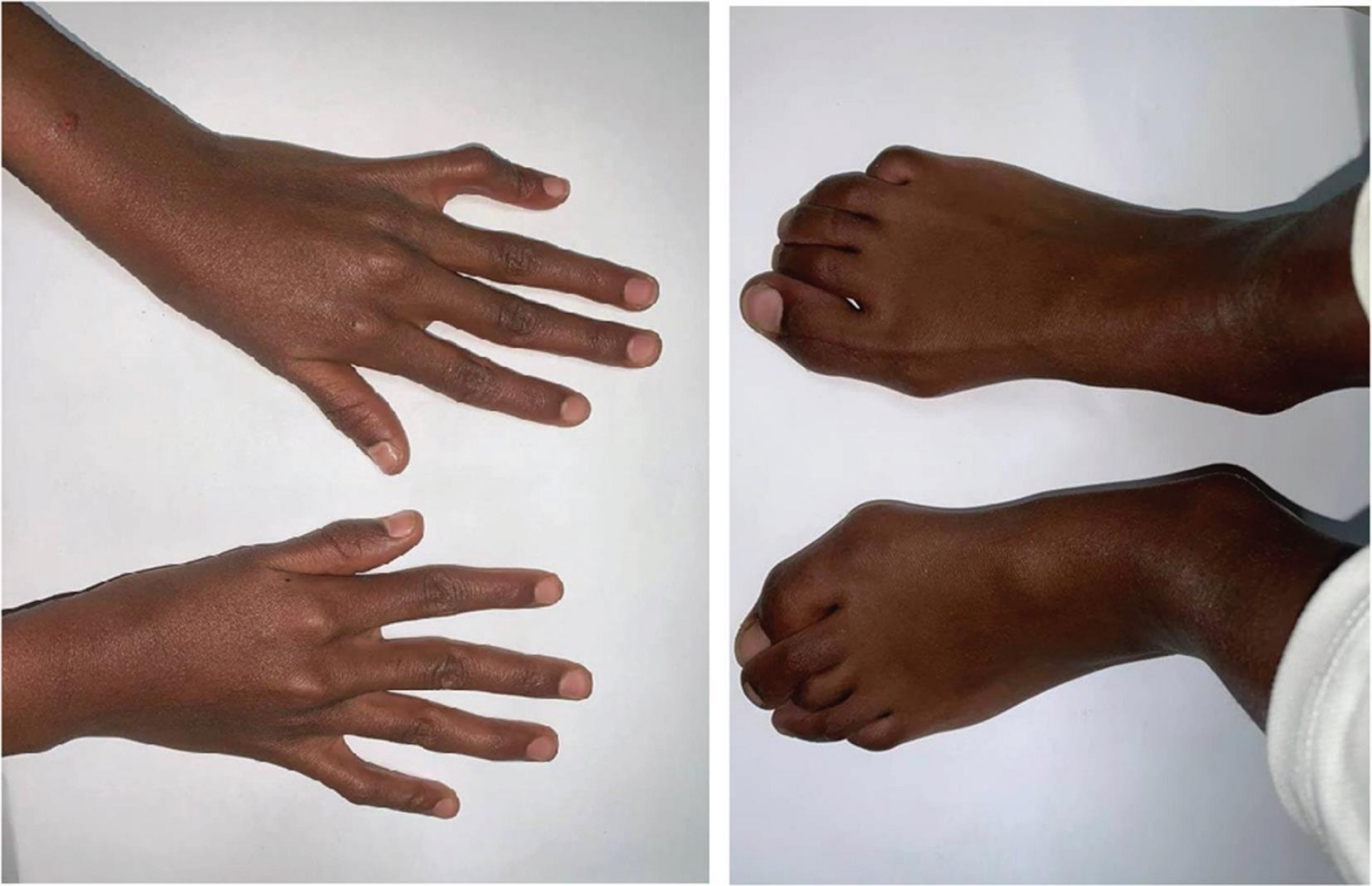
Bilateral camptodactyly, mild ulnar deviation of the fingers, and valgus deformity of the big toes bilaterally.

**Figure 3:**
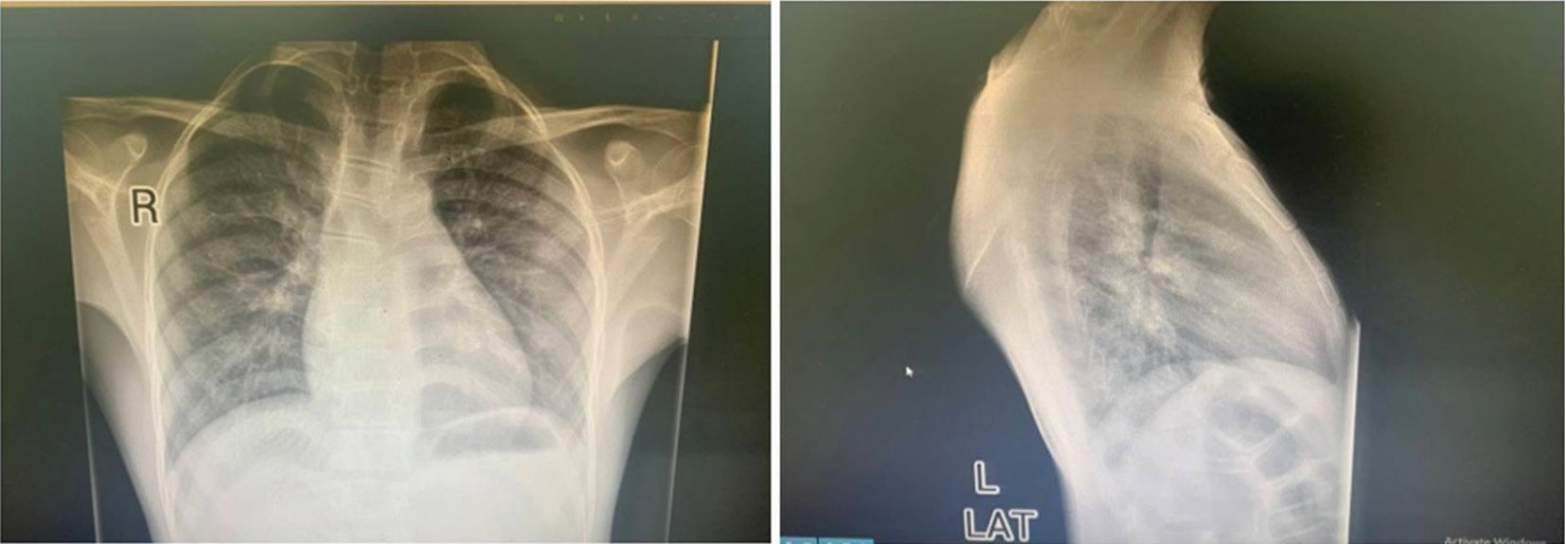
PA and lateral chest X‐rays show thoracic mild scoliosis.

Routine noninvasive cardiac and pulmonary screenings were negative. Musculoskeletal evaluation disclosed a spinal column exhibiting loss of thoracic kyphosis, mild ulnar deviation of the fingers, bilateral camptodactyly, and hallux abducto valgus bilaterally, as illustrated in Figure 2. Electrocardiography (ECG), echocardiography, and laboratory analyses yielded normal results. Chest radiographs indicated mild thoracic scoliosis (see Figure 3).

### 2.2. Management

#### 2.2.1. Surgical Technique: Bilateral Oral Commissuroplasty Using Converse′s Technique

**Figure 4:**
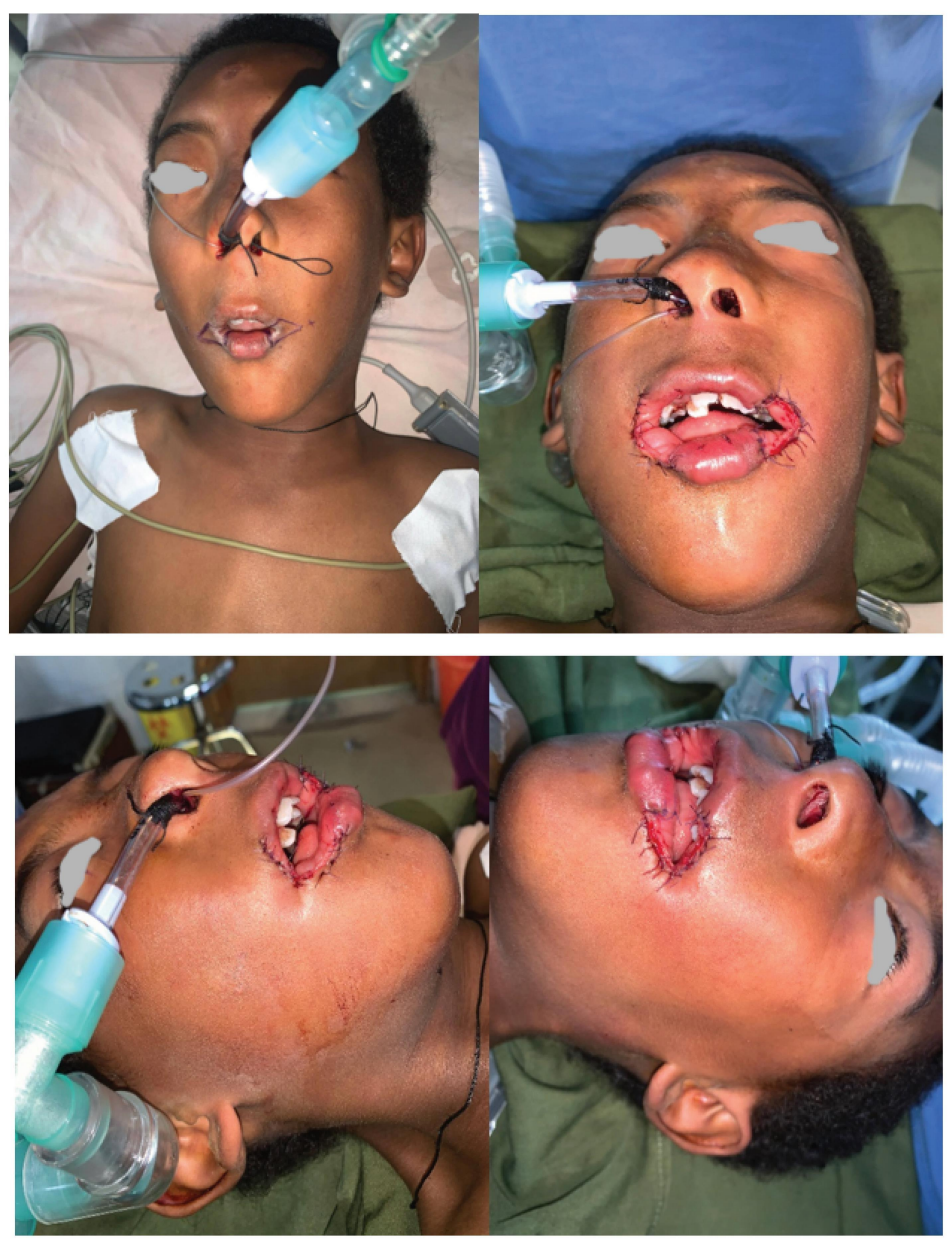
Intraoperative pictures showing skin marking and neocommissure created with Converse′s V‐Y mucosal advancement technique.

**Figure 5:**
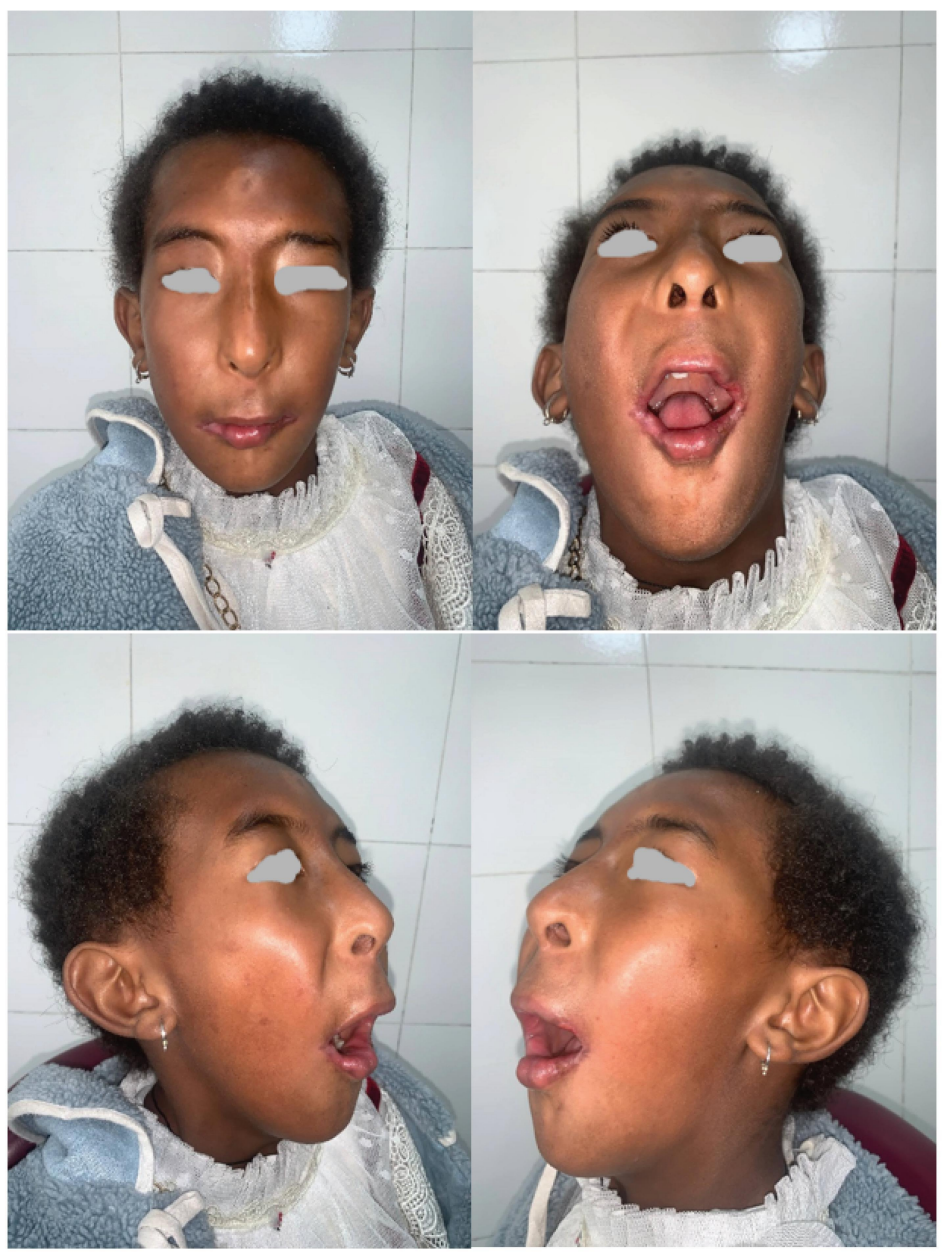
Postoperative patient pictures 1 month after surgery.

After fiber‐optic assisted nasal intubation, the neocommissure was marked lateral to the existing commissure, leveled to a hypothetical perpendicular line passing through the mid‐pupil, as shown in Figure 4. The site was then prepped in standard fashion with iodine‐based antiseptic. Following local infiltration of lidocaine mixed with epinephrine along the incision line, bilateral triangular facial skin incisions were made with a No. 15 blade. These incisions had their base at the blunted commissure and apex at the neocommissure. Full‐thickness facial skin was then resected, creating a V‐shaped skin defect. The remaining buccal mucosa and the labial mucosa of the blunted commissure were divided horizontally at the midline, extending to the neocommissure point, thereby creating two triangular mucosal flaps. The divided upper and lower triangular labial and buccal mucosa were advanced and secured to the facial skin superiorly and inferiorly, respectively, using polyglycolic acid suture. The upper and lower lips were lengthened by 10 mm on each side, creating a new commissure and enlarging the intercommissural dimension to approximately 40 mm, as shown in Figure 5.

The extraoral and intraoral sutures were removed on postoperative Days 7 and 15, respectively. Figure 5 shows the patient′s condition at 1 month postoperatively. Adequate lengthening of the horizontal axis of the mouth and enlargement of the oral stoma were achieved. This improved oral access for feeding and enhanced the ability to perform dental hygiene.

#### 2.2.2. Anesthesia‐Related Concerns and Management

Preoperatively, the anesthesia team, surgeons, and nursing staff were advised of the patient′s FBS diagnosis and its anesthesia‐related challenges. A difficult airway was anticipated due to microstomia, limited neck extension, and potential temporomandibular joint involvement. Out of an abundance of caution, an MH anesthetic protocol was followed, including use of a clean anesthesia machine purged with 100% oxygen before use and avoidance of volatile anesthetic agents (e.g., halothane, sevoflurane, and isoflurane) and succinylcholine.

After nebulizing with 3 mL of lidocaine for 15 min to decrease airway reactivity and promote mucus clearance, the patient was taken to the operating room. Intraoperatively, a total intravenous anesthesia technique using propofol infusion was induced, and fiber‐optic‐assisted nasal intubation was performed. Standard monitoring, including ECG, pulse oximetry, noninvasive blood pressure monitoring, continuous capnography, and core temperature monitoring via a nasopharyngeal probe, were used. The patient remained calm and hemodynamically stable throughout the procedure, which was completed uneventfully and lasted 90 min. She was then awakened from anesthesia, extubated, and transferred to the postanesthesia care unit (PACU) with stable vital signs.

Postoperatively, extended monitoring was continued in the PACU and subsequently on the inpatient ward. Pain was managed with nonopioid analgesics to prevent respiratory depression. The patient was discharged on the second postoperative day.

## 3. Discussion

FBS, previously referred to as Freeman–Sheldon syndrome, represents a congenital myopathic condition affecting the craniofacial region. Most characteristically, FBS presents with a small “whistling” mouth, a flattened mask‐like face, club feet, and joint contractures. Although extracranial features like club feet and joint contractures are commonly observed, they are nonspecific findings and are not required to establish the diagnosis. FBS was first documented by Freeman and Sheldon in 1938 [12] and later independently recognized as a distinct entity by Burian in 1963, who introduced the evocative term “whistling face” [7]. Transmission follows an autosomal dominant pattern, although the majority of cases arise sporadically without any documented family history [13]. Craniofacial deformities, including the severely restricted oral aperture, interfere with eating, speech articulation, dental hygiene, and airway access. Additionally, the characteristic whistling appearance imposes a substantial psychological burden [14].

FBS can result from allelic variations in the embryonic myosin heavy chain gene, situated on Chromosome 17 at Band 17p13.1 [15]. The most frequently observed variations appear to disrupt adenosine triphosphate binding to myosin. This disruption is thought to influence muscle physiology during early development, attenuating muscle growth and leading to residual muscle contractures [8, 15]. In patients with FBS, surgical findings reveal white fibrous tissue interspersed within normal muscle fibers, as well as complete replacement of muscle by fibrous and adipose tissue [16]. Some muscles remain grossly and histologically normal [16]. These surgical observations align with in vitro studies showing anomalies in the metabolic processes required for contraction and extreme muscle stiffness that reduces muscular work and power output [16–19]. Electromyography data have confirmed contracture of the orbicularis oris, masseter, buccinator, and risorius muscles, along with contraction of the temporomandibular joint capsule [20].

FBS is an exceptionally uncommon disorder whose true prevalence remains unknown, largely because of diagnostic uncertainty [21]. Far fewer than 100 published cases have actually satisfied the strict diagnostic criteria for FBS [8, 22]. The syndrome appears to exhibit no predilection for gender, ethnicity, or geographic region, and environmental or parental factors have not been implicated in its pathogenesis [13, 21].

The diagnostic criteria for FBS are based on physical examination findings, and additional testing does not contribute to confirming a diagnosis but is required for treatment planning. The diagnostic criteria [15, 23, 24] necessitate the presence of the following features: microstomia; a whistling‐face phenotype characterized by pursed lips; a defect in the chin that is either H‐ or V‐shaped; and pronounced nasolabial folds. Diagnostic criteria differ on the inclusion of significant contractures affecting two or more anatomical regions, typically the hands and feet, but patients meeting the craniofacial part of the criteria have historically been diagnosed with the syndrome. The limb malformations include talipes equinovarus, metatarsus varus, vertical talus, talipes equinovalgus, calcaneovalgus, camptodactyly, ulnar deviation of the wrists and fingers, overlapping digits, and hypoplastic or absent interphalangeal creases [15, 17, 23]. Frequently, spinal deformities, gastroenterological complications, and various craniofacial anomalies are observed [23]. FBS is commonly misidentified with other conditions that exhibit phenotypic similarities yet are genotypically and histologically distinct [18, 23, 25]. The differential diagnosis presents considerable challenges owing to the syndrome′s heterogeneous clinical manifestations. Numerous disorders may exhibit features reminiscent of FBS, including distal arthrogryposis types 1A, 1B, 2B, 3, 7, and 8; Schwartz–Jampel syndrome; and nonsyndromic distal contractures [17, 18, 23].

Treating microstomia, whether congenital or acquired, is consistently challenging. In every instance, the difficulty lies in preserving function while achieving acceptable aesthetics. Various nonsurgical and surgical treatment methods have been described for managing microstomia [1, 26, 27].

Nonsurgical approaches include oral physiotherapy with orthotic devices, pulsed light therapy, hyaluronidase, and botulinum toxin injections [27, 28]. These techniques are preferred for mild cases and offer advantages such as simplicity and lower morbidity compared with surgical approaches. However, they demonstrate limited effectiveness in patients with moderate to severe microstomia [1, 29]. Different static and dynamic orthotic devices can be employed to provide resistance to scar contraction or to promote stretching and mobility, respectively. Static devices, such as commissural splints, help maintain perioral tissues in good condition during the healing period. To be effective, they must be easy to design, relatively comfortable for the patient, and effective at preventing tissue contraction [1, 30]. Dynamic appliances are classified into three categories according to the type of stretching provided (horizontal, vertical, and circumoral) and are further grouped based on whether the device is intraoral or extraoral, and unilateral or bilateral. Intraoral devices may be fixed or removable, and both types can be customized [26, 30, 31]. The appliances used for contracture management in FBS range from passive pressure stents to high‐tension retractors. The response to therapy varies with patient compliance and the extent of scar tissue present [1, 32].

The surgical reconstruction of the oral cavity and the perioral regions is frequently characterized by significant complexity attributing to the intricate transition of the vermilion border, the circular action of the orbicularis oris muscle, and the absence of an underlying bony structure to provide support [33, 34]. Throughout the years, a variety of surgical techniques were devised to address this challenge, each geared toward achieving an equilibrium between aesthetic naturalness and functional efficacy. The available techniques encompass a spectrum that includes skin grafts and local flaps, extending to more intricate free tissue transfers sourced from alternative anatomical regions. The determination of the most appropriate approach is fundamentally contingent upon several critical considerations: the patient demographics, the degree of microstomia, and the location and depth of the scarring, as well as the condition of the local vascular supply [2]. In instances where microstomia is less complex and the scarring is predominantly superficial, skin grafts are often effective, particularly following the release of the contracted tissue [2, 35]. In cases characterized by moderate microstomia accompanied by deeper fibrotic changes or in instances of congenital microstomia, local tissue rearrangements frequently become pertinent. This may involve mucosal flaps such as the V‐Y advancement, rhomboid, or trapeze configurations, along with skin‐based techniques including double Z‐plasty, asterisk flaps, or nasolabial pedicle flaps [36, 37]. In patients with severe microstomia where considerable tissue loss has adversely affected the local blood supply, free tissue transfer is often regarded as the optimal, and at times the sole, recourse. In such scenarios, surgeons may resort to fasciocutaneous flaps, such as the anterolateral thigh flap or the superficial circumflex iliac artery perforator flaps, or innervated muscle flaps, such as the gracilis or latissimus dorsi, to facilitate not only the restoration of bulk but also the reinstatement of movement and functionality [38–41].

Amelioration of microstomia in FBS is not merely a cosmetic procedure; it constitutes a functional reconstruction, and reoperation may be necessary as fibrous bands can reform. The aim is to achieve an oral aperture that improves oral intake. Several authors have reported different surgical interventions to improve microstomia in FBS. The bilateral V‐Y mucosal advancement flap, originally described by Dieffenbach and later modified by Converse, is the workhorse procedure for primary FBS microstomia. It recruits redundant buccal mucosa, increases the horizontal dimension of the oral opening, and preserves the natural vermilion border. However, it carries risks of scarring, potential webbing at the commissures, altered sensation, and the need for postoperative therapy to adapt to the new oral dynamics [42]. Ferreira and colleagues reported sectioning the orbicularis oris muscle to lengthen the oral aperture and using a U‐shaped skin incision at the mouth corner to create an adequate corner; the vermilion deficit of the upper and lower lips was then reconstructed using mucosal advancement flaps [43]. Neumann and Coetzee noted that the upper lip is aesthetically more important and used lower lip vermilion to correct upper lip vermilion deficiency [44].

The anesthetic management of patients with FBS presents several challenges. It is complicated by orofacial contractures, limited neck mobility, spinal deformities, and difficult vascular access [11]. Although there is no evidence that true MH was associated with FBS, some still suggest using an MH‐safe anesthetic technique when possible [11].

The most common challenge is airway management, as severe microstomia, micrognathia, immobile orofacial musculature, Class II malocclusion, dental crowding, a highly arched palate, and limited cervical spine flexibility may make endotracheal intubation and the use of airway adjuncts difficult [11]. Several case reports have outlined strategies for airway management in FBS, emphasizing difficulties with intubation and the various methods used to manage the difficult airway in these patients [45, 46]. Although some clinicians might consider using a laryngeal mask airway (LMA) as a strategy to circumvent the difficulties of endotracheal intubation, LMAs are generally not viewed as a suitable airway management choice for this patient group. The combination of a tiny oral opening, restricted mouth aperture, and specific oropharyngeal anatomy often prevents proper insertion and adequate seating of an LMA, increasing the risk for GERD [47]. Instead, fiber‐optic bronchoscopy represents the preferred technique for securing the airway. In emergency situations or cases of exceptional intubation difficulty, surgical airway like cricothyroidotomy or tracheotomy may be required; however, this procedure itself can be technically challenging owing to underdeveloped cartilaginous structures [11]. Despite successful administration of anesthesia to FBS patients, intraoperative masseter muscle spasm and generalized muscle rigidity after induction have been reported [10, 48]. Moreover, even after an uneventful intraoperative course, postoperative morbidity and mortality due to atelectasis, bronchitis, or upper airway tract obstruction have been reported by many authors [49].

Other challenges associated with FBS may include profoundly difficult vascular access due to distal extremity contractures and the consequent poor quality of veins, requiring use of a small‐gauge catheter (22 gauge or smaller). The need for such a small catheter may impair transfusion, intravenous hydration, medication administration, and blood draw efforts. This may necessitate the use of ultrasound‐assisted peripheral vein cannulation or central line placement to provide vascular access for these patients [11].

## 4. Conclusion

FBS is not a progressive condition. Feeding challenges in these patients are multifactorial and poorly understood, largely because of a historical lack of investigation. Optimal outcomes depend on an accurate and early diagnosis, an understanding of the myopathic craniofacial basis and origin of the syndrome, and an anticipatory and coordinated team‐based strategy. Management must specifically target the fibrous constricting bands, ensure an adequate oral aperture for nutrition and dental care, support long‐term needs of the patient, and incorporate careful preparation for anesthesia‐associated complications.

## Funding

No funding was received for this manuscript.

## Consent

Written informed consent was obtained from the family of the patient about the use of clinical data and accompanying patient images in the publication of this case report.

## Conflicts of Interest

The authors declare no conflicts of interest.

## Data Availability

The data that support the findings of this study are available on request from the corresponding author. The data are not publicly available due to privacy or ethical restrictions.
